# Mismatch Negativity and P50 Sensory Gating in Abstinent Former Cannabis Users

**DOI:** 10.1155/2016/6526437

**Published:** 2016-02-25

**Authors:** Samantha J. Broyd, Lisa-marie Greenwood, Hendrika H. van Hell, Rodney J. Croft, Hannah Coyle, Ben Lee-Bates, Juanita Todd, Stuart J. Johnstone, Patricia T. Michie, Nadia Solowij

**Affiliations:** ^1^School of Psychology, University of Wollongong, Wollongong, NSW 2522, Australia; ^2^Illawarra Health and Medical Research Institute, University of Wollongong, Wollongong, NSW 2522, Australia; ^3^Centre for Health Initiatives, University of Wollongong, Wollongong, NSW 2522, Australia; ^4^School of Psychology and Priority Research Centre for Translational Neuroscience and Mental Health, University of Newcastle, Newcastle, NSW 2308, Australia; ^5^Schizophrenia Research Institute, Sydney, NSW 2021, Australia

## Abstract

Prolonged heavy exposure to cannabis is associated with impaired cognition and brain functional and structural alterations. We recently reported attenuated mismatch negativity (MMN) and altered P50 sensory gating in chronic cannabis users. This study investigated the extent of brain functional recovery (indexed by MMN and P50) in chronic users after cessation of use. Eighteen ex-users (median 13.5 years prior regular use; median 3.5 years abstinence) and 18 nonusers completed (1) a multifeature oddball task with duration, frequency, and intensity deviants and (2) a P50 paired-click paradigm. Trend level smaller duration MMN amplitude and larger P50 ratios (indicative of poorer sensory gating) were observed in ex-users compared to controls. Poorer P50 gating correlated with prior duration of cannabis use. Duration of abstinence was positively correlated with duration MMN amplitude, even after controlling for age and duration of cannabis use. Impaired sensory gating and attenuated MMN amplitude tended to persist in ex-users after prolonged cessation of use, suggesting a lack of full recovery. An association with prolonged duration of prior cannabis use may indicate persistent cannabis-related alterations to P50 sensory gating. Greater reductions in MMN amplitude with increasing abstinence (positive correlation) may be related to either self-medication or an accelerated aging process.

## 1. Introduction

Regular and prolonged cannabis use is associated with a range of adverse psychological outcomes, including psychosis [1–4]. The primary constituent of cannabis, Δ^9^-tetrahydrocannabinol (THC), is a partial agonist at central cannabinoid receptor (CB1R) sites which are densely located throughout the brain but especially within regions critical to attention, learning, and memory, such as the hippocampus, amygdala, and prefrontal cortex [5]. It is these cognitive functions in particular that are impaired in cannabis users (for a review, see [6]), arguably due to cannabis-related alterations to the regulatory role that the endocannabinoid system plays in synaptic plasticity [7–10]. The latter is thought to occur, at least in part, via downregulation of N-methyl-D-aspartate (NMDA) receptor activity [7–9, 11]. Evidence is also emerging for analogous alterations in cannabis users and patients with schizophrenia, including cognition, brain function, and structure, and there is increasing support for an association between cannabis and schizophrenia in vulnerable individuals [12–14]. In light of this evidence, research has turned to examining common neurobiological and neuropsychological markers of dysfunction (for a review, see [6]).

Two such candidate markers are the mismatch negativity (MMN) and the P50, event-related potential (ERP) brain markers of early sensory memory and of sensory gating, respectively. MMN is sensitive to NMDA receptor function (for a review, see [15, 16]) and elicited by irregular acoustic deviations (“deviants”) in a predictable pattern of auditory stimuli (called standards [17, 18]) reflecting evaluation of new auditory sensory input against current models of the acoustic environment [18]. Robust reductions in MMN have been noted in patients with schizophrenia [19, 20] and less consistently in their first degree relatives [21–23]. Cannabis users also show reduced MMN, with the strongest evidence for alterations to frequency deviants [24–26], but three studies have also reported group differences for duration deviants [24, 27, 28]. We recently reported significantly smaller frequency MMN in both long-term and short-term users compared to controls, whereas duration MMN was reduced only in long-term users, and duration MMN amplitude reduction was associated with the duration of regular and, in particular, daily cannabis use [24]. Consistent with mounting evidence of an association between cannabis use and risk for psychosis, reductions in duration and frequency MMN were associated with a retrospective measure of psychotic-like symptoms during intoxication [24].

P50 sensory gating is also impaired in both patients with schizophrenia [29, 30] and chronic cannabis users [31–34]. Sensory gating refers to the brain's ability to regulate its response to incoming stimuli [35] or to “gate out” irrelevant information from further processing [36–38]. (Sensory gating-“out” may be contrasted with the brain's ability to “gate in” novel or new information, of which the MMN is a marker [36, 37].) P50 sensory gating is impaired in regular cannabis users compared to both nonuser controls [31–34] and patients with schizophrenia with and without comorbid cannabis use [33]. Longer duration of use [33] and greater quantity of monthly use [32] were associated with more pronounced P50 gating impairments.

The existence of brain alterations in cannabis users leads to the important question of whether these are amenable to recovery following cessation of cannabis use. Persistent IQ decline has been reported in adult cannabis users who started using cannabis during adolescence and who had recently abstained or significantly reduced cannabis use for at least one year [39]. Results from more sensitive neuroimaging studies of users with at least 25 days abstinence indicate persistent alterations to brain function [40–46]. We previously reported impaired ERP measures of selective attention in former users with a mean 2-year abstinence that were associated with duration of prior use [47]. Pertinent to the current study, Rentzsch et al. [33] identified persistent deficits in P50 sensory gating in cannabis users who had been abstinent for at least 28 days and in a subsequent study [25] persistent reductions in frequency MMN amplitude in former users with a mean 19-month abstinence. Neither study investigated the association between duration of abstinence or prior cannabis use and recovery of brain function. Therefore, although these findings suggest persistent cannabis-related alterations to MMN and P50 sensory gating following cessation of cannabis use, further research is required to replicate these findings and determine the degree to which recovery may occur as a function of prior cannabis exposure and length of abstinence.

The aim of the current cross-sectional study, in which we recruited former cannabis users with a range of abstinence and prior history of use, was to investigate (1) whether MMN amplitude and P50 sensory gating improve as a function of increasing periods of abstinence following cessation of use and (2) the extent to which recovery may depend on the degree of prior cannabis use. Consistent with previous work, we predicted that ex-users would exhibit attenuated frequency and duration MMN (impaired sensory processing) and larger P50 ratios and smaller P50 difference scores (poorer sensory gating) compared to nonuser controls and that improvement in brain function would be positively correlated with duration of abstinence and negatively correlated with duration of prior cannabis use.

## 2. Materials and Methods

### 2.1. Participants

Twenty abstinent former cannabis users and 42 healthy nonuser controls took part in the current study. Ex-cannabis users were required to have used cannabis regularly (≥3 times/week) for at least two years prior to quitting and to have abstained for at least one month; two were excluded for recent other drug use leaving a sample of 18 ex-users. Nonuser control participants were required to have <20 lifetime occasions of cannabis use and none in the previous 12 months. Recruitment and exclusion criteria are described in Supplement 1 in Supplementary Material available online at http://dx.doi.org/10.1155/2016/6526437. Controls were recruited as part of a larger study (see [24, 31]). Whilst ex-users completed all experimental paradigms, not all controls completed both MMN and P50 paradigms. Therefore, two overlapping subsamples (*n* = 18 each) of the most appropriately age-matched controls were blindly selected for MMN and P50 analyses, respectively, from the nonuser sample reported in Greenwood et al. [24] (12 participants were included in both control groups). All participants were reimbursed AUD$50 for their participation. The study was approved by the University of Wollongong and Illawarra Shoalhaven Local Health District Health and Medical Human Research Ethics Committee.

### 2.2. Demographic and Substance Use Variables

Participants underwent a comprehensive structured interview to assess demographic information, detailed history of current and previous substance use, and psychiatric history and ex-users provided a urine sample to verify recent abstinence. Participants were excluded if they reported a personal or familial (first degree relative) history of any psychotic disorder. Participants reported (i) the last time they used any cannabis and how much; (ii) their previous patterns of regular use (i.e., their usual pattern of use, periods when they used cannabis more or less frequently, and periods of abstinence); and (iii) when they stopped using cannabis regularly. This informed two key variables to define the duration of abstinence: (i) time since last use, defined in years since last use of any cannabis and (ii) time since last regular use of cannabis (> once/month for any 6-month period).

The vocabulary and matrices subscales of the Wechsler Abbreviated Scale of Intelligence (WASI [49]) were used to estimate full scale IQ. The Rey Auditory Verbal Learning Test (RAVLT) was also administered and participants completed the Community Assessment of Psychic Experiences (CAPE [50]) and Schizotypal Personality Questionnaire (SPQ [51]) to assess psychotic-like symptoms, the Beck Depression Inventory (BDI [52]), State Trait Anxiety Inventory (STAI [53]), Edinburgh Handedness Inventory [54], and the Alcohol Use Disorders Identification Test (AUDIT [55]). Ex-cannabis users also completed the Marijuana Withdrawal Checklist (MWC [56]), Severity of Dependence Scale (SDS [57]), and Cannabis Experiences Questionnaire (CEQ, [58]) to measure symptoms of withdrawal and retrospective severity of dependence and symptoms experienced whilst intoxicated.

### 2.3. Experimental Paradigms

The passive multifeature MMN paradigm and recording conditions, with duration (100 ms), frequency (1200 Hz), and intensity (90 dB; standards 50 ms, 1000 Hz, 80 dB) deviants, are described in Greenwood et al. [24] and further in Supplement 1. For the MMN task participants were instructed to ignore the tones and focus their attention on a silent film. The attended P50 paired-click paradigm (in which participants were asked to silently count the click pairs and respond to every 25th click pair via button press) and recording condition (ISI 9 seconds) were identical to the paradigm described in Broyd et al. [31] and in Supplement 1. Participants were seated in a comfortable chair approximately 80 cm away from a monitor in a dimly lit room. All stimuli were presented binaurally using headphones (Sennheiser HD215). Measurement details of MMN and P50 are described in Supplement 1 and Greenwood et al. [24] and Broyd et al. [31] respectively.

### 2.4. Statistical Analysis

To investigate potential group differences on demographic factors (age, gender, and IQ), psychological symptoms, verbal learning, and alcohol and tobacco use, independent sample *t*-tests or Mann-Whitney *U* tests (for data not normally distributed) were employed. Group comparisons of MMN peak amplitude were performed using repeated measures Analysis of Variance (rmANOVA) including a within-subject variable, condition (3: duration, frequency, and intensity deviant), and between-subject factor, group (2: control and ex-user). P50 ratio and difference scores were compared between groups using Mann-Whitney *U* tests as the data were not normally distributed. Correlational analysis investigated associations between MMN and P50 measures, cannabis measures (duration of abstinence and of prior use), and relevant demographic, clinical, and cognitive measures in the ex-user sample. As prior research suggests that frequency and duration MMN are reduced in cannabis users in particular [24], correlations between MMN and cannabis use history including duration of abstinence were restricted to these deviant conditions.

## 3. Results

### 3.1. Cannabis Use History and Abstinence

Ex-users had previously used cannabis regularly for a median of 13.5 years at approximately 6 joints/day on a median of 30 days/month (see Table 1). They had not used cannabis regularly for a median of 3.5 years and had been totally abstinent (since last occasion of any use) for a median of 1.5 years (range of 6 weeks to 16 years for both measures of abstinence). No participants had used any substance other than alcohol or tobacco regularly (defined as more than once/month for a 6-month period) in the past 5 years.

### 3.2. Group Comparisons on Demographic and Clinical Variables

No differences between ex-users and controls were identified for age, gender, handedness, verbal learning (i.e., RAVLT performance), or symptom measures (Table 2) other than the SPQ scores reported below.

*MMN Group Comparisons*. Ex-users had fewer years of education (*Z* = −3.64, *p* < .001), tended to have lower IQ (*Z* = −1.80, *p* = .073), consumed a greater quantity of alcohol (*Z* = −2.08, *p* = .04) with a trend toward greater frequency (*Z* = −1.71, *p* = .09), and smoked more cigarettes than controls (*Z* = −3.00, *p* = .003). Ex-users had elevated SPQ social anxiety (*Z* = −2.03, *p* = .04) and a trend toward elevated SPQ total (*Z* = −1.74, *p* = .08) scores. None of these variables were correlated with MMN amplitude in any deviant condition and therefore none were included as covariates in any further analyses (see Supplement 1).

*P50 Group Comparisons.* Ex-users tended to have lower IQ scores (*Z* = −1.87, *p* = .064) and had fewer years of education (*Z* = −3.92, *p* < .001) than controls. They smoked more cigarettes per day (*Z* = −2.97, *p* = .01) but did not differ from controls in quantity or frequency of alcohol use (*p* > .10). Ex-users again had elevated SPQ social anxiety (*Z* = −2.43, *p* = .015) and a trend towards elevated SPQ total (*Z* = −1.76, *p* = .079) scores. None of these variables were correlated with P50 metrics and therefore none were included as covariates in any further analyses (see Supplement 1).

### 3.3. Mismatch Negativity in Ex-Users and Controls

**Figure 1:**
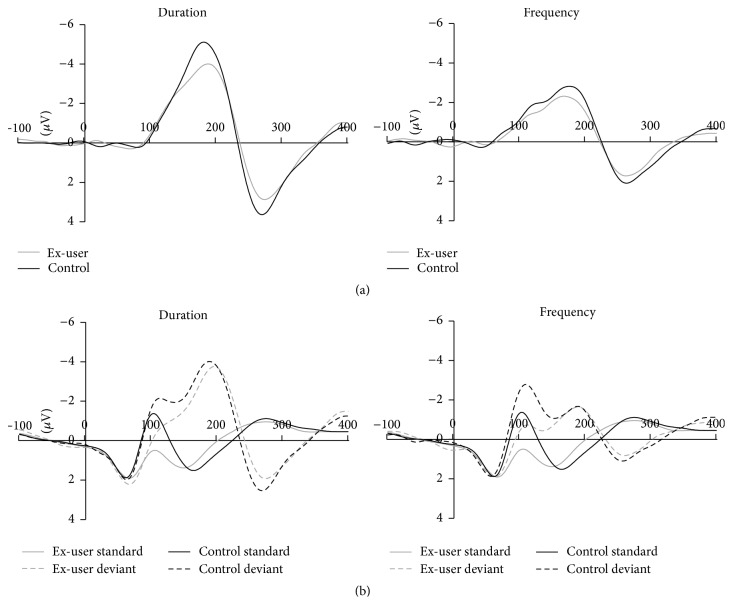
(a) Mismatch negativity (MMN) waveforms at Fz displayed for each condition for ex-cannabis users (grey line) and nonuser controls (black line). (b) Mastoid reference unsubtracted ERP waveforms at Fz to standard (solid line) and deviant tones (dashed lines) for ex-cannabis users (grey lines) and nonuser controls (black lines). Amplitude is shown in *μ*V on the *y*-axis and time in milliseconds along the *x*-axis.

Mismatch negativity difference waveforms at Fz for duration and frequency conditions are shown in Figure 1(a) and grand mean ERP waveforms at Fz to the standard and deviant stimuli are shown in Figure 1(b). Despite visible group differences in the standard ERP around N1 (explored in Supplement 1), there were no significant differences between ex-users and controls in mean amplitude for standard ERPs over the latency windows used for MMN peak detection.

**Figure 2:**
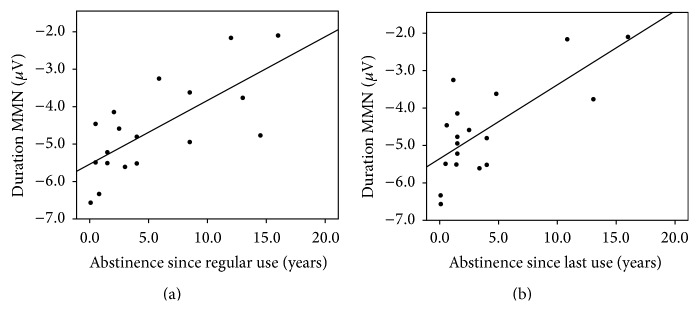
Scatter plots showing association between duration MMN amplitude (*μ*V) and duration of abstinence since last regular cannabis use (a) and abstinence since the last occasion of use (b).

Ex-users had visibly smaller MMN amplitudes than controls, although this did not reach significance as a main effect of group (*F*(1,34) = 1.02, *p* = .32). However, there was a main effect of condition (*F*(2,68) = 38.63, *p* < .001) and a significant condition by group interaction (*F*(2,68) = 3.34, *p* = .042) (Table 3(a)). Univariate ANOVAs were used to decompose the significant condition × group interaction and compared ex-users and controls in each deviant condition separately. MMN in each deviant condition revealed no effect of group for frequency MMN (*F*(1,34) = 1.99, *p* = .17) or intensity MMN (*F* < 1.0), although groups differed at trend level for duration MMN (*F*(1,34) = 2.97, *p* = .09; *p* = .08 with age included as a covariate (see Supplement 1)) with smaller duration MMN amplitudes in ex-users. Age of onset of regular use, duration of regular and daily use, and quantity and frequency of prior cannabis use were not significantly correlated with either frequency or duration MMN amplitude (all *p* > .09). However, duration MMN was positively correlated with abstinence (time since regular use: *ρ* = .65, *p* = .004; time since last use: *ρ* = .56, *p* = .016), with the pattern of results not appreciably affected by controlling for either duration of use (partial *r* = .72, *p* = .001 and *r* = .74, *p* = .001, resp.) or age (partial *r* = .63, *p* = .007 and *r* = .65, *p* = .005, resp.) separately or combined (partial *r* = .42, *p* = .053 and *r* = .47, *p* = .032, resp.). However, age and duration of use were not correlated with duration MMN after controlling for time since regular use (age: partial *r* = −.10, *p* = .72; duration of regular use: partial *r* = .05, *p* = .85) or time since last use (age: partial *r* = .10, *p* = .69; duration of regular use: partial *r* = .13, *ρ* = .61). These results indicate smaller duration MMN with longer periods of abstinence (Figure 2).

### 3.4. P50 Sensory Gating in Ex-Users and Controls

**Figure 3:**
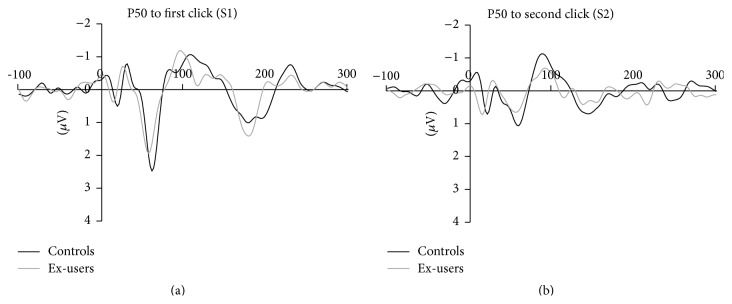
ERP waveforms to the first (S1; (a)) and second (S2; (b)) click at Cz for ex-cannabis users (grey) and nonuser controls (black). Amplitude is shown in *μ*V on the *y*-axis and time in milliseconds along the *x*-axis.

Grand mean ERP waveforms at Cz to the first (S1) and second (S2) click are presented for ex-users and controls in Figure 3, and P50 amplitudes to S1 and S2, P50 ratio, and difference scores in Table 3(b). Ex-users did not differ from controls in terms of P50 difference score (*Z* = −1.23, *p* = .23). There was a trend toward larger P50 ratios in ex-users compared to controls (*Z* = −1.68, *p* = .097).

**Figure 4:**
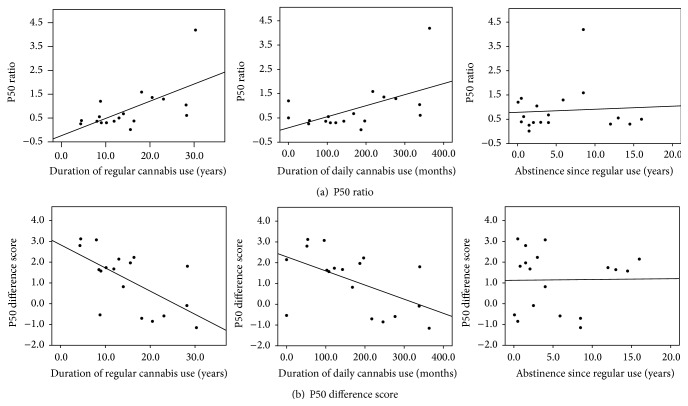
Scatter plots showing association between P50 metrics and duration of regular cannabis use in years (left), duration of daily cannabis use in months (middle), and duration of abstinence since regular use (right) for ex-cannabis users. P50 ratio shown on the top row (a) and P50 difference score shown on the bottom row (b).

In ex-users, no correlation was observed between age of onset of regular cannabis use, quantity or frequency of prior cannabis use per month, time since last regular use or time since last use, and either P50 ratio or difference score (all *p* > .10). However, duration of prior regular use and duration of prior daily use were both significantly correlated with P50 ratio (regular use: *ρ* = .61, *p* = .007; daily use: *ρ* = .50, *p* = .033, Figure 4) and P50 difference score (regular use: *ρ* = −.60, *p* = .008; daily use: *ρ* = −.55, *p* = .019, Figure 4). These correlations remained significant after controlling for the duration of abstinence since regular use in partial correlations (P50 ratio, regular use: *r* = .63, *p* = .006; daily use: *r* = .57, *p* = .02; P50 difference score, regular use: *r* = −.64, *p* = .006; daily use: *r* = −.55, *p* = .02). Further, time since last regular use or time since last occasion of any use was not correlated with P50 metrics after controlling for duration of regular or daily use (time since regular use: all *p* > .3; time since last use: all *p* > .6).

## 4. Discussion

Given mounting evidence of altered MMN and P50 sensory gating in chronic cannabis users, the current study set out to examine the degree to which impairments in early sensory processing might recover with abstinence from cannabis, as a function of the duration of abstinence and history of prior use. We report trends toward impaired sensory gating (larger P50 ratios) and reduced duration MMN amplitude in ex-cannabis users compared to nonuser controls. An association between the duration of prior cannabis use and P50 metrics suggests that persistent impairments in P50 gating may be related to prior exposure to cannabis. In contrast, the association between MMN amplitude and duration of abstinence was not in the expected direction, with smaller amplitudes associated with longer abstinence. The P50 data extend the findings of Rentzsch et al. [33] who found that P50 sensory gating was reduced in former cannabis users who were abstinent for at least 28 days: the ex-cannabis users of the present study had been abstinent for a median of 3.5 years (range of 6 weeks to 16 years). The observed association between greater P50 impairment and duration of prior regular and daily cannabis use in this study, and in Rentzsch et al. [33], and no association with the duration of abstinence, highlights the possibility that these persistent effects may result from prolonged exposure to cannabis and may not recover with abstinence. The findings concur also with previous research in current cannabis users (with 12–24 hours of abstinence to control for acute effects [31, 32, 34, 59]), implicating an association between cannabis exposure and functioning of neuronal generators involved in sensory gating. Unlike Rentzsch et al. [25], the current study did not find frequency MMN amplitude to be reduced in ex-users relative to controls.

There is increasing evidence that regular exposure to cannabis over an extended period of time is associated with reduced frequency MMN [24–26] and more recently, in long-term users, attenuated duration MMN as well [24]. We have argued that reduction in duration MMN may manifest only after protracted (and especially daily) cannabis use. We suggested that attenuated frequency MMN in short- and long-term users may be related to altered gyrification and cortical thinning in temporal and frontal regions observed to be unrelated to the extent of exposure to cannabis in users [60], while duration MMN may be sensitive to broader alterations to brain function associated with sound duration processing following more prolonged exposure to cannabis [24]. In the current study of former users, we did not observe an association between duration of regular (or daily) cannabis use and attenuated duration MMN, although, unexpectedly, we found that longer durations of abstinence (both since last regular use and since the last occasion of use) were correlated with greater reductions in duration MMN amplitude. This relationship is difficult to interpret, as it is not possible in the current sample to definitively dissociate aging from length of abstinence (or duration of cannabis exposure). Nevertheless, partial correlations controlling for age and for duration of prior regular use revealed that age and duration of prior regular use did not account for this relationship. This association speaks to three possible hypotheses: first, that the association between duration MMN and abstinence may reflect* accelerated* aging, with persistent and nonlinear effects beyond cessation of cannabis use in former users. Second, it is possible that individuals who have used cannabis and subsequently chosen to abstain differ fundamentally from nonusing individuals on a third unmeasured variable for which duration of abstinence is acting as proxy. Nevertheless further analysis (reported in Supplement 1) suggests that duration of abstinence was not correlated with neuropsychological functioning, alcohol or cigarette use, psychosis-proneness, or prior cannabis use. A third hypothesis may therefore be that cannabis use may have “medicated” a preexisting deficit in former users and that this deficit is progressively unmasked with ongoing abstinence. That duration MMN, previously demonstrated by us to correlate with longer duration of cannabis use (particularly daily use) [24], suggests that any potential preexisting deficit was nevertheless exacerbated by long-term cannabis use, perhaps interacting with the aging process. Currently speculative, further research is required to (dis)confirm these hypotheses.

The precise mechanisms by which prolonged exposure to cannabis might alter the neuronal substrates which underpin P50 indices of sensory gating remain unclear [61]. The generators of the P50 evoked potential have been localised to Heschl's gyrus in the primary auditory cortex [62–64]. Less clear are the neurobiological substrates involved in sensory gating [63]. Currently it is thought that inhibitory inputs from the CA3 region within the hippocampus, whilst not directly related to P50 generation [65], may act to suppress activity in the primary auditory cortex linked with P50 generation to the second click [63]. Potential generators in the frontal lobe [63] and superior temporal gyrus [66] have also been implicated, in addition to several neurotransmitter systems including dopaminergic, serotonergic, and glutamatergic systems [67]. A possibility, therefore, is that THC impacts upon inhibitory inputs from the hippocampus via densely located CB1 receptors alter an individual's sensory gating capacity. Furthermore, as we have suggested previously [31], there may be a threshold of exposure associated with the duration of regular or daily cannabis use over which impairments in sensory gating arise and this may affect the degree to which these deficits persist after cessation of use and similarly the degree to which they recover. Future research might take a longitudinal approach to further examine P50 metrics before and after cessation of cannabis use, to assess potential changes over time within the same individuals.

There are a number of limitations in the current study which may be addressed by future work. First, as a cross-sectional and naturalistic study we relied on participant self-report for the duration of abstinence and history of cannabis use. The current study set out to examine MMN and P50 metrics as a function of a broad duration of abstinence and prior use, and for this reason it was not possible to supervise abstinence in a way consistent with protocols employed in prior research examining short durations of abstinence (e.g., 28 days) [68]. As such, the duration of abstinence in this sample was broadly distributed, but more than half had abstained for the period between 6 weeks and 5 years, which should have captured any evidence for either abrupt or gradual recovery during the early period following cessation of use, as well as enabling detection of evidence for any gradual recovery that might require a significant period of abstinence. No evidence for any such patterns was observed; however, this requires replication in a larger sample of ex-cannabis users. Second, despite our observation that altered P50 metrics were significantly associated with the duration of exposure to cannabis, the extent to which these alterations to brain function are present prior to cannabis exposure remains unknown and may be answered by longitudinal studies. Third, we acknowledge an argument that while education and IQ did not correlate with our ERP measures and were therefore inappropriate as covariates in our analyses, the group differences on these measures may point to differences in cognitive function that could affect potential recovery or even predate cannabis use. This cannot be easily ruled out. Nevertheless, ex-cannabis users in the current study did not differ from nonuser controls in terms of verbal learning and memory performance, and there were very few differences between groups in terms of psychological wellbeing, including psychosis-proneness. Future research should examine the functional significance of these apparently persistent alterations in sensory gating in long-term former cannabis users and the extent to which they may impact upon functioning in daily life and/or predate cannabis use affecting recovery of function. We acknowledge that the sample size of the ex-user group was small. Nevertheless, the sample represents a unique population of clean cannabis users (unconfounded by other drug use or psychopathology) with long histories of prior cannabis use and with prolonged abstinence, a sample that is difficult to recruit and is rarely reported in the literature.

## 5. Conclusions

In summary, the trend level pattern of results of the current study suggests that cannabis-related alterations to MMN and P50 may not fully recover following cessation of long-term cannabis use. Instead, the current data suggest that (i) prolonged exposure to cannabis alters P50 sensory gating and these alterations may persist beyond cessation of use and (ii) MMN amplitude may reduce further with increasing abstinence. The results raise the possibility of persistent alterations to the regulatory role of the endocannabinoid system on brain function, affecting the brain's ability to regulate its response to incoming stimuli or to filter irrelevant information. These results require replication in future studies of brain functional plasticity-related recovery in abstinent cannabis users.

## Supplementary Material

Supplement 1 contains information pertaining to (i) recruitment and exclusion criteria; (ii) mismatch negativity and P50 task and measurement details; (iii) exploratory analysis of N1 amplitude to standard and deviant stimuli; (iv) analysis of P50 to S1 and S2 stimuli separately; (v) outlier analyses; and (vi) exploratory correlation analyses between MMN and P50, and demographic and clinical measures and verbal learning.

## Figures and Tables

**Table 1:** Cannabis use measures including abstinence, retrospective measures of dependence and psychological symptoms during intoxication, and withdrawal symptoms.

	Ex-cannabis users
Prior cannabis use	
Frequency (days/month)	30 [15.0–30.0]
Quantity (cones^a^/month)	517.5 [52.5–3150.0]
Age of onset (years)	17 [12.0–20.5]
Duration of regular use (years)	13.5 [4.3–30.3]
Duration of daily use (months)	155.3 [0.0–363.7]
Time since last smoked (years)	1.5 [0.1–16.0]
Abstinence since regular use (years)	3.5 [0.1–16.0]
SDS^b^	8.50 [1.0–13.0]
CEQ^c^	
Euphoria	40 [32.0–52.0]
Paranoia	48 [28.0–87.0]
After effects	27 [15.0–51.0]
Amotivation	18.5 [10.0–31.0]
Psychotic	8 [4.0–20.0]
MWC^d^	10.5 [0.0–37.0]

*Notes*. Data are reported as median [range].

^a^Cones used in waterpipe: 3 cones are equivalent to one standard sized joint [48].

^b^Severity of Dependence Scores (SDS) (retrospective)

^c^Cannabis Experiences Questionnaire (CEQ) (retrospective)

^d^Marijuana Withdrawal Checklist (MWC) (retrospective).

**Table 2:** Demographic, cognitive, and psychological symptom measures in ex-cannabis users and healthy nonuser controls for MMN and P50 analyses.

	Ex-cannabis users	MMN controls	P50 controls
	(*n* = 18)	(*n* = 18)	(*n* = 18)
Age (years)	39.1 [20.8–56.0]	40.4 [21.0–52.6]	31.2 [20.1–52.6]
Gender (male/female)	10 M/8 F	10 M/8 F	9 M/9 F
Handedness	All right-handed	Two left-handed	All right-handed
Education (years)	12.0 [10.0–16.0]	14.3 [11.5–20.0]^*∗∗*^	14.5 [11.5–20.0]^*∗∗*^
IQ	105.0 [89.0–126.0]	115.5 [89.0–133.0]^*Ϯ*^	115.0 [89.0–133.0]^*Ϯ*^
RAVLT trials I to V	54.00 [34.0–68.0]	54.5 [29.0–69.0]	56.50 [29.0–70.0]
RAVLT trial VI	11.5 [5.0–15.0]	12.0 [4.0–15.0]	12.0 [4.0–15.0]
RAVLT trial VII	11.5 [0.0–15.0]	11.0 [3.0–15.0]	11.0 [3.0–15.0]
Alcohol frequency^a^	3.5 [0.0–30.0]	1.0 [0.0–12.0]^*Ϯ*^	1.4 [0.0–12.0]
Alcohol quantity^b^	16.8 [0.0–180.0]	5.1 [0.0–28.0]^*∗*^	6.3 [0.0–70.0]
Cigarettes (per day)	3.8 [0.0–35.0]	0.0 [0.0–12.0]^*∗*^	0.0 [0.0–11.0]^*∗*^
Psychological symptoms			
K10	15.0 [12.0–31.0]	14.0 [10.0–19.0]	14.0 [10.0–22.0]
BDI	6.5 [0.0–18.0]	3.0 [0.0–19.0]	3.0 [0.0–19.0]
STAI-I	31.0 [20.0–67.0]	32.0 [20.0–45.0]	27.5 [20.0–45.0]
STAI-II	39.0 [21.0–62.0]	36.0 [22.0–50.0]	32.5 [20.0–45.0]
CAPE			
Frequency total	58.0 [46.0–76.0]	60.0 [46.0–89.0]	59.0 [47.0–89.0]
Distress total	20.0 [4.0–51.0]	25.5 [6.0–95.0]	22.0 [5.0–50.0]
Negative frequency	21.5 [16.0–35.0]	21.5 [12.0–31.0]	21.0 [15.0–31.0]
Negative distress	11.0 [2.0–30.0]	10.5 [2.0–41.0]	9.0 [2.0–22.0]
Positive frequency	23.5 [20.0–31.0]	25.0 [21.0–44.0]	26.0 [20.0–44.0]
Positive distress	5.5 [0.0–20.0]	6.0 [1.0–29.0]	6.0 [0.0–18.0]
Depressive frequency	12.5 [9.0–16.0]	13.0 [9.0–21.0]	12.0 [9.0–21.0]
Depressive distress	6.0 [2.0–15.0]	8.0 [2.0–25.0]	7.0 [1.0–21.0]
SPQ			
Total	18.0 [9.0–32.0]	14.0 [5.0–32.0]^*Ϯ*^	12.0 [2.0–39.0]^*Ϯ*^
Ideas of reference	1.0 [0.0–6.0]	1.0 [0.0–4.0]	1.0 [0.0–5.0]
Social anxiety	3.0 [0.0–15.0]	2.0 [0.0–6.0]^*∗*^	1.0 [0.0–6.0]^*∗*^
Odd beliefs	1.0 [0.0–5.0]	0.0 [0.0–5.0]	0.0 [0.0–7.0]
Unusual perceptual	1.0 [0.0–3.0]	1.0 [0.0–2.0]	1.0 [0.0–4.0]
Odd behaviour	2.0 [0.0–5.0]	0.0 [0.0–6.0]	0.0 [0.0–6.0]
Close friends	2.0 [0.0–8.0]	1.0 [0.0–3.0]	1.0 [0.0–4.0]
Odd speech	4.0 [0.0–7.0]	4.0 [1.0–8.0]	3.0 [0.0–8.0]
Constricted affect	1.0 [0.0–5.0]	1.0 [0.0–2.0]	1.0 [0.0–4.0]
Suspiciousness	1.0 [0.0–5.0]	1.0 [0.0–3.0]	1.0 [0.0–3.0]

*Notes*. Comparisons between ex-cannabis users and respective nonuser controls for each paradigm (MMN or P50); ^*Ϯ*^ *p* < .10, ^*∗*^ *p* < .05,  ^*∗∗*^ *p* < .001. Data reported as median [range].

^a^Alcohol frequency measured as number of days per month alcohol was consumed.

^b^Alcohol quantity measured as number of standard drinks consumed per month.

RAVLT: Rey Auditory Verbal Learning Test; K10: Kessler Psychological Distress Scale; BDI: Beck Depression Inventory; STAI-I: State Anxiety Index State score; STAI-II: State Anxiety Index Trait score; CAPE: Community Assessment of Psychic Experiences; SPQ: Schizotypal Personality Questionnaire.

**Table 3:** (a) Mean (SD) MMN amplitude (*μ*V) and latency (ms) at Fz and (b) median [range] P50 metrics (*μ*V) at Cz for ex-cannabis users and healthy nonuser controls.

	Ex-cannabis users (*n* = 18)	Controls (*n* = 18)
(a) MMN		
Duration MMN		
Amplitude (*μ*V)	−4.60 (1.26)	−5.65 (2.26)
Latency (ms)	183.9 (20.6)	183.8 (17.2)
Frequency MMN		
Amplitude (*μ*V)	−2.74 (1.21)	−3.34 (1.35)
Latency (ms)	167.6 (22.2)	173.1 (21.7)
Intensity MMN		
Amplitude (*μ*V)	−3.25 (1.51)	−2.92 (1.87)
Latency (ms)	153.6 (27.6)	142.4 (25.9)
(b) P50		
P50 S1 amplitude	2.59 [0.36–5.14]	2.80 [0.39–9.52]
P50 S2 amplitude	1.83 [0.03–3.20]	1.27 [0.0–3.62]
P50 ratio	0.53 [0.01–4.19]	0.37 [0.0–1.41]
P50 difference score	1.66 [−1.15–3.12]	1.83 [−0.40–5.90]
