# Supplementary material for: Mismatch Negativity and P50 Sensory Gating in Abstinent Former Cannabis Users
Source: Neural Plast. 2016 Feb 25;2016:6526437. doi: 10.1155/2016/6526437 (PMC4785272; doi:10.1155/2016/6526437)
Supplement: Supplementary file 1 — Supplement 1 contains information pertaining to (i) recruitment and exclusion criteria; (ii) mismatch negativity and P50 task and measurement details; (iii) exploratory analysis of N1 amplitude to standard and deviant stimuli; (iv) analysis of P50 to S1 and S2 stimuli separately; (v) outlier analyses; and (vi) exploratory correlation analyses between MMN and P50, and demographic and clinical measures and verbal learning. [file 6526437.f1.pdf]

Mismatch negativity and P50 sensory gating in abstinent former cannabis users

Supplemental Section

Detailed Methods

Inclusion and exclusion criteria for participants

All participants were recruited via advertisements in local newspapers, and were screened for inclusion and exclusion criteria over the telephone before making an appointment. Participants were required not to have used any other illicit substance regularly (> monthly for more than 6 months) in the last 2 years, and to abstain from using alcohol (or any substance other than tobacco) for at least 12 hours prior to testing. Abstinence from other illicit drugs was corroborated by urinalysis. Two participants in the ex-cannabis group were excluded due to recent substance use other than cannabis. No participant had used any other drug regularly in the past 5 years, although two participants reported prior regular substance use more than 5 years ago: one had used ecstasy regularly for a period of 6 months, 5 years ago; and a second reported frequent hallucinogen use (mushrooms) for a period of 5 years, 10 years ago. Participants were also required to abstain from caffeinated drinks and nicotine for the duration of the testing procedure: EEG recording commenced a minimum of 1.5 hours into the testing schedule. The telephone screening interview included assessing for the possibility of a current psychiatric condition using the Kessler Psychological Distress Scale (K10, 1). Two participants in the ex-user group had a score of 30 or over on the K10 indicating a high level of risk of mental disorder. Both participants also had a score of 18 on the BDI indicating mild depression, however no participants reported any current or previous diagnosis or treatment for any psychological disorder during a face to face structured interview conducted prior to EEG protocols. The primary analyses were repeated excluding the two participants with prior regular substance use and the two with high K10 scores, as described below.

Procedure

All participants were first familiarised with the study procedures before written informed consent was obtained. Prior to commencing the EEG protocols, all participants also completed audiometric testing and were found to have hearing within the normal range (i.e. ≤ 25 dB at 1000Hz and 1500Hz). Participants were then fitted with electrodes and completed the following tasks in order: an eye-movement calibration task to be used in subsequent ocular artifact correction procedures (2), the multi-feature MMN paradigm (3) and the P50 paired-click paradigm (4).

Multi-feature MMN paradigm: In this paradigm, standard stimuli (presented on 82% of trials) were 1000Hz tones, 80dB SPL and 50ms duration, with a 5ms rise and fall time. Three types of deviant stimuli (each with a probability of 6%) were presented in a pseudorandom sequence with the standard stimulus (at least 2 and a maximum of 7 standard

stimuli separated each of the deviant stimuli): a duration deviant (100ms), frequency deviant (1200Hz) and an intensity deviant (90dB SPL).

P50 paired click paradigm: This task consisted of one block of 50 pairs of 1ms click stimuli presented at 90 dB SPL in a random fashion on average 9 seconds apart (3 slightly different IPI lengths were used to give the appearance of randomness, the mean of which was the target IPI: e.g. 8930, 8990 or 9080ms), with the first and second click pairs separated by a fixed-inter-stimulus interval of 500ms.

Electrophysiological data acquisition and analysis

Electroencephalographic (EEG) data were recorded continuously from 19 Ag/AgCl electrodes positioned on an electrode cap according to the international 10-20 system, and two electrodes positioned over the left and right mastoid. EEG data were grounded to an electrode placed midway between FPz and Fz, and referenced online to an electrode positioned on the tip of the nose. Four monopolar electrodes were placed above and below the left eye and 1 cm from outer canthi of the left and right eye and used to calculate vertical, horizontal and radial electrooculogram (EOG) data required for the EOG correction procedure of Croft and Barry (2). Data were sampled at 500 Hz, with a bandpass filter of 0.1 to 100 Hz. All electrode impedances were below 10 kΩ at the start of recording.

Mismatch negativity analysis:

EEG data were analysed offline using Neuroscan software (Scan 4.4), and were re-referenced to the average of the mastoids and corrected for ocular artifacts according to Croft and Barry (2000). EEG data were bandpass filtered from 1 to 30Hz (down 24 dB/octave roll off), epoched from 100 ms pre-stimulus to 400 ms post-stimulus, and baseline corrected using the 100ms pre-stimulus interval. Epochs containing EEG data exceeding ± 50 μV were rejected. EEG data were then averaged to the standard tone and each of the deviant conditions (duration, frequency and intensity) separately. For each deviant type, MMN difference waveforms were computed by subtracting the event-related potential (ERP) waveform for the standard stimuli from each of the ERP waveforms for the deviant stimuli. The MMN waveforms were then lowpass filtered at 20Hz (down 24 dB, see 5).

MMN peak amplitude and latency analyses were restricted to Fz only (6, 7) consistent with Greenwood et al., (3). MMN peak amplitude at Fz was identified as the most negative peak 130-230ms post-stimulus for duration deviants and 100-200ms post-stimulus for frequency and intensity deviants, according to the following criteria: nose-referenced MMN data must display a frontocentral negative topography (94% of participants met this criterion) and a polarity reversal at the mastoids (71% of participants met this criterion). MMN data from all participants met at least one of these criteria and were therefore retained in subsequent analyses.

For the analysis of N1 to standards and deviants, the largest negative peak between 70 and 130 ms at Fz was identified using an automatic peak detection algorithm and then verified visually by an experienced researcher.

P50 sensory gating analysis:

EEG data were analysed offline using Neuroscan software (Scan 4.4), and were bandpass filtered from 10 to 45 Hz (down 12 dB/octave roll off), epoched from 100 ms pre-stimulus to 300 ms post-stimulus, and baseline corrected using the 100 ms pre-stimulus interval. EEG data were epoched to the first (S1) and second (S2) click, and were rejected if signals at any EEG channel exceeded ± 50 µV.

P50 peak amplitude was measured at Cz in accordance with Clementz et al.(8). P50 peaks were first identified using an automatic peak detection algorithm and then verified visually by an experienced researcher. P50 peak amplitude to S1 was measured relative to the preceding peak (Na; 48±15 ms) and identified as the largest positive peak around the average P50 peak latency (i.e. 62±15 ms) with a central topography (4). P50 peak amplitude to S2 was measured relative to the preceding peak, with the additional criteria that (Na and P50) latency must fall within 20ms of an individual's S1 peak latency. All participants met these criteria. There was no minimum amplitude required to be selected as a peak. P50 was recorded to the first (S1) and second (S2) click, and two measures of sensory gating were computed: P50 ratio, defined as the ratio of P50 peak amplitudes (i.e. S2/S1, where smaller P50 ratios are indicative of better sensory gating); and P50 difference score, defined as the difference between P50 peak amplitude to the first and second click (i.e. S1- S2, where larger difference scores are indicative of better sensory gating).

Statistical analysis

Following visual inspection of the ERP waveforms to standard and deviant stimuli in the MMN multi-feature paradigm, which suggested that N1 amplitude may be attenuated in ex-users relative to controls, N1 amplitude to standard and deviant stimuli was also analysed. Group comparisons of N1 peak amplitude were performed using a repeated measures Analysis of Variance (rmANOVA) with stimulus type as the within-subjects factor (standard and duration, frequency and intensity deviants) and group (control, ex-user) as the between-subjects factor.

MMN amplitude and P50 metrics (P50 amplitude to S1, S2, P50 ratio and difference score) were examined for outliers (more than ±1.5 times the inter-quartile range) and analyses were repeated with and without identified outliers excluded. Where the pattern of effects remained unchanged following exclusion of outliers, analyses with all cases included are reported in the main paper. Where the pattern of results changed following the exclusion of outliers, the data are reported without outliers included below.

Primary MMN and P50 analyses were also rerun following (i) exclusion of two ex-users with a period of regular polysubstance use more than 5 years ago, and (ii) two ex-users with a K10 score over 30 (see *1.1 Inclusion and Exclusion criteria for participants* above). Where the pattern of effects remained unchanged following the exclusion of these participants, analyses with all cases included were reported in the main paper. Where the pattern of results changed following the exclusion of these participants, the analyses are reported without these participants below.

In addition to the primary analyses reported within the main paper, Spearman's correlations were used to explore associations between MMN amplitude and P50 metrics (P50 ratio and difference score) and psychosis proneness (CAPE, SPQ) and verbal learning in the ex-user groups.

Finally, below we report group comparisons for P50 amplitude to S1 and S2 using Mann Whitney-U tests as the data were not normally distributed.

Additional Results

N1 amplitude in ex-cannabis users and controls to deviant and standard stimuli in a multi-feature MMN task

Grand mean ERP waveforms at Fz to the standard and deviant stimuli are shown in Figure 1b in the main paper and mean (SD) N1 amplitude reported in Table S1. The rmANOVA revealed a main effect of group with significantly reduced N1 amplitude in ex-users compared to controls ($F(1,34)=8.45$, $p=.006$) but no stimulus type by group interaction ($F<1$). Further, a main effect of stimulus type ($F(3,34)=27.74$, $p<.001$) revealed larger N1 amplitudes in each deviant condition compared to the standard stimulus (all $p<.001$), and a larger N1 to intensity compared to duration deviants ($p=.001$). In the absence of a group difference in the standard waveform during the MMN window (see main paper), these results may indicate earlier alterations to feature encoding and stimulus perception in ex-users (9). Nevertheless, future research should consider a larger montage to ascertain whether these differences in N1 reflect in sensory specific or non-specific components of the N1(9).

Outlier analyses and analyses (i) excluding 2 participants with regular polysubstance use more than 5 years ago, (ii) excluding 2 participants with K10 scores over 30.

MMN analysis: No outliers were identified in the control group or ex-user group. When MMN analyses were rerun with 2 participants with high K10 scores excluded, the trend level reduction in MMN amplitude in ex-users compared to controls was rendered non-significant ($p = .146$). The patterns of effects for all other analyses remained unchanged. The exclusion of two participants with a history of polysubstance use more than 5 years ago did not change the pattern of effects for the main analysis.

P50 analysis: For the main analysis of ex-users and controls, the trend toward larger P50 ratios in ex-users compared to controls became significant after exclusion of two control and one ex-user outliers (Z=-2.02, p=.045), indicating impaired sensory gating in the ex-users. The exclusion of two participants with high K10 scores and separately, two participants with a history of polysubstance use more than 5 years ago did not change the pattern of effects for the main analysis.

Associations between MMN and demographic, clinical measures and verbal learning

Age did not differ between groups and age was not correlated with MMN amplitude in any condition in the ex-user group (all p>.14). In the control group age was correlated with frequency MMN amplitude (p=.02), but not duration MMN (p=.25) or intensity MMN (p=.07). Analyses were repeated with age included as a covariate, however the pattern of results remained unchanged and the trend difference between groups for duration MMN amplitude strengthened (p<.079).

No association for either ex-users or controls was identified between MMN amplitude (in any condition) and current IQ (*controls:* all p>.16; *ex-users:* all p>.47), K10 (*controls:* all p>.64; *ex-users:* all p>.58), BDI (*controls:* all p>.30; *ex-users:* all p>.46), STAI state (*controls:* all p>.32; *ex-users:* all p>.26), STAI trait (*controls:* all p>.33; *ex-users:* all p>.47), alcohol frequency and quantity (*controls:* all p>.42; *ex-users:* all p>.11), cigarettes smoked per day (*controls:* all p>.05; *ex-users:* all p>.40), or verbal learning performance (total words recalled, recall after interference and recall after a delay; *controls:* all p>.07; *ex-users:* all p>.09).

Psychosis-proneness was examined in ex-users only, consistent with the study aims. No significant correlations were observed between MMN in ex-users and CAPE total frequency and distress scores or any CAPE subscale scores (all p>.20), nor SPQ total score (all p>.12). Frequency MMN amplitude was, however, positively associated with the Suspiciousness (ρ(17)=.55, p=.02) and Odd Speech subscales (ρ(17)=.53, p=.03) of the SPQ, indicating greater reductions in MMN amplitude with higher scores, consistent with recent proposals that MMN may be a useful marker of psychosis risk (3, 7). Duration MMN was negatively correlated with the Constricted Affect subscale (ρ(17)=-.55, p=.02) indicating larger MMN amplitudes with higher scores. However, these associations were no longer significant after applying Bonferonni corrections.

To examine whether duration of abstinence may be acting as a proxy for a third variable (given the strong association between duration of abstinence and MMN), we explored correlations between duration of abstinence, neuropsychological functioning, alcohol and cigarette use, psychosis-proneness or prior cannabis use. Abstinence was not correlated with any variable (all p>.10) except age (time since last regular use: *(ρ(17)*=.493, p=.038; time last smoked: *(ρ(17)*=.438, p=.069) and the Odd or Eccentric subscale of the SPQ (time since last regular use: *(ρ(17)*=.511, p=.036; time last smoked: *(ρ(17)*=.495, p=.043). Nevertheless, after correcting for multiple tests, none of these

associations remained significant. Furthermore, neither age (all p>.14) nor the Odd or Eccentric subscale of the SPQ were correlated with frequency or duration MMN (all p>.17). In summary, whilst it is still possible that duration of abstinence is acting as a proxy for an unmeasured third variable, it was not correlated with neuropsychological functioning, alcohol and cigarette use, psychosis-proneness or prior cannabis use in our ex-user sample. Therefore these data support greater reductions in MMN with increasing years abstinence and, in combination with trend level findings of attenuated MMN amplitude in ex-users, are suggestive of an accelerated aging or self-medication hypothesis.

Associations between P50 and demographic, clinical measures and verbal learning

In the control group, no significant associations were observed between P50 metrics (ratio, difference score), and IQ (all p>.14), K10 (all p>.44), BDI (all p>.68), STAI-state (all p>.36), STAI-trait (all p>.54), alcohol quantity (all p>.11), alcohol frequency (all p>.09), cigarettes smoked per day (all p>.10), or verbal learning (all p>.32).

In the ex-user sample, no significant associations were observed between P50 metrics and IQ (p>.39), K10 (all p>.36), BDI (all p>.24), STAI-trait (all p>.63), alcohol frequency (all p>.18), cigarette use per day (all p>.50), verbal learning (all p>.70), CAPE total frequency or quantity (or any subscale; all p> .09) nor SPQ total or subscale (all p> .08). In ex-users, the P50 difference score, but not ratio (p=.19), was positively correlated with STAI-state anxiety (($\rho(17)$=.61, p>.007), indicating larger difference scores with greater state anxiety. P50 ratio was also negatively correlated with alcohol quantity in ex-users (P50 ratio, ($\rho(17)$=-.48, p=.042; but not P50 difference score, ($\rho(17)$=.44, p=.069) although the groups did not differ on alcohol consumption, which was relatively low level in the sample, and these associations did not survive correction for multiple comparisons.

P50 amplitude to S1 and S2 analysis in ex-users and controls

Grand mean ERP waveforms at Cz to the first (S1) and second (S2) click are presented for ex-users and controls in Figure 3 and P50 amplitudes to S1 and S2, P50 ratio and difference score in Table 3b of the main paper. Ex-users did not differ from controls in P50 amplitude to S1 (Z=-.13, p=.91) or P50 amplitude to S2 (Z=-1.36, p=.18). In ex-users, no correlation was observed between age of onset of regular cannabis use, quantity or frequency of prior cannabis use per month, duration of abstinence since regular use or time since last occasion of use and P50 S1 or S2 amplitude (all p>.05).

Duration of regular prior cannabis use and duration of daily prior cannabis use were both significantly correlated with P50 amplitude to S1 (regular use: ρ=-.61, p=.007; daily use: ρ=-.61, p=.007). These correlations remained significant after controlling for the duration of abstinence since regular use in partial correlations (regular use: r=-.53, p=.03; daily use: r=-.56, p=.02). Further, time since last regular use or time since last occasion of any use were not correlated with P50 metrics after controlling for duration of regular use (since regular use: all p>.4; since last use: all

p>.6) or duration of daily use (since regular use: all *p*>.3; since last use: all *p*>.6). Consistent with correlations between prior exposure and P50 ratio and difference metrics, this suggests early sensory gating processes may not recover following cessation of use.

Supplemental References

1. Andrews G, Slade T (2001): Interpreting Scores on the Kessler Psychological Distress Scale (K10). *Aust N Z J Public Health* 25:494-497.
2. Croft RJ, Barry RJ (2000): Eog Correction: Which Regression Should We Use? *Psychophysiology* 37:123-125.
3. Greenwood LM, Broyd SJ, Croft R, Todd J, Michie PT, Johnstone S, et al. (2014): Chronic Effects of Cannabis Use on the Auditory Mismatch Negativity. *Biol Psychiatry* 75:449-458.
4. Broyd SJ, Greenwood LM, Croft RJ, Dalecki A, Todd J, Michie PT, et al. (2013): Chronic Effects of Cannabis on Sensory Gating. *Int J Psychophysiol* 89:381-389.
5. Kujala T, Tervaniemi M, Schröger E (2007): The Mismatch Negativity in Cognitive and Clinical Neuroscience: Theoretical and Methodological Considerations. *Biol Psychol* 74:1-19.
6. Duncan CC, Barry RJ, Connolly JF, Fischer C, Michie PT, Näätänen R, et al. (2009): Event-Related Potentials in Clinical Research: Guidelines for Eliciting, Recording and Quantifying Mismatch Negativity, P300, and N400. *Clin Neurophysiol* 120:1883-1908.
7. Umbricht D, Krljes S (2005): Mismatch Negativity in Schizophrenia: A Meta-Analysis. *Schizophr Res* 76:1-23.
8. Clementz BA, Geyer MA, Braff DL (1998): Multiple Site Evaluation of P50 Suppression among Schizophrenia and Normal Comparison Subjects. *Schizophr Res* 30:71-80.
9. Naatanen R, Kujala T, Winkler I (2011): Auditory Processing That Leads to Conscious Perception: A Unique Window to Central Auditory Processing Opened by the Mismatch Negativity and Related Responses. *Psychophysiology* 48:4-22.

Table S1.

Mean (SD) N1 amplitude measured in microvolts (μV) and latency in milliseconds (ms) at Fz in ex-cannabis users and healthy non-user controls.

	**Ex- users (n=18)**	**Controls (n=18)**
Standard (μV)	0.48 (1.19)	-1.50 (1.57)
Standard (ms)	106.0 (13.6)	104.7 (6.1)
Duration (μV)	-0.92 (1.31)	-2.36 (1.95)
Duration (ms)	109.4 (13.3)	115.4 (10.9)
Frequency (μV)	-1.19 (1.37)	-3.06 (2.18)
Frequency (ms)	107.9 (9.0)	111.7 (8.4)
Intensity (μV)	-2.06 (1.70)	-3.19 (2.31)
Intensity (ms)	112.8 (9.4)	111.6 (10.7)
